# Cerebro-Spinal Meningitis in Bristol

**Published:** 1940

**Authors:** B. A. Peters

**Affiliations:** Medical Superintendent, Ham Green Hospital, Bristol


### The Bristol Medico-Chirurgical Journal

*"Scire est nescire, nisi id me
Scire alius sciret."*

SUMMER, 1940.

### CEREBRO-SPINAL MENINGITIS IN BRISTOL.

BY

B. A. Peters, M.D., D.P.H.,

*Medical Superintendent, Ham Green Hospital, Bristol.*

Since the beginning of January, 1940, forty-two cases of bacteriologically proven cases of meningococcal meningitis have been admitted to Ham Green Hospital.

The age incidence was relatively highest under 5, as twelve cases fell in this group; ten in the 5-10 age-group; eleven between 10 and 20; and only nine over 20. The outbreak, compared with the last large outbreak in 1915, appears to be about the same severity, as twenty cases were very acutely ill, with delirium, restlessness and loss of consciousness; nineteen were moderately ill, with severe headache but no loss of consciousness; and three showed comparatively mild symptoms. A very unusual feature of these cases was the complete absence of pyrexia in sixteen of them, irrespective of the severity of the attack.

Exactly half of the cases had rashes of a purpuric nature. In the majority the purpuric spots were only 1 to 2 mm. in diameter, but four cases showed large purpuric blotches, in one case 10 cm. in diameter. As regards the distribution of the rash, it was most marked on the back, but was generally present on the limbs and, in a few cases, spots were present on the face. Five others showed small macular pink rashes, very strongly resembling the rash of rubella; in fact, two of the cases were admitted with this diagnosis. Four had marked facial herpes, and the remainder no rash whatever.

A greater or lesser degree of neck rigidity was present in every case, and Kernig's sign could also be elicited in every case. Signs of cranial nerve involvement were observed in two cases only: one had a temporary left ptosis and another case a temporary diplopia. Two other cases showed non-suppurative arthritis involving the ankle-joints. The remainder were completely free from any complications, and made perfect recoveries with no residual damage up to the time of discharge, which was generally four to six weeks from the onset.

One unusual case occurred in an adult, not included in the above series. From the culture of the cerebro-spinal fluid a few meningococci and a large number of pneumococci, Type III, were grown. This case ended fatally.

The cases were all treated with sulphapyridine in the following doses: for adults one gramme was given two-hourly for three doses, then four-hourly up to the end of the fourth day, when half the dose was continued for two further days. For younger patients the dose was calculated on the following basis, as children seem more tolerant of the drug than adults: children were given the proportion of the above doses based on their weight relative to eight stone; that is to say that a child of four stone was given half this dose. With these doses and for the period stated no marked effect was found on the leucocyte count.

In many cases vomiting due to the increased intracranial pressure was a very troublesome symptom. For these the soluble sodium salt was given intramuscularly in the same doses until the vomiting ceased, when it was continued by mouth. It is very important to give this drug intramuscularly, in which site it appears to produce very little inconvenience. If given subcutaneously, it causes a localized necrosis of the tissues and a punched-out ulcer which is very slow in healing. This is apparently due to the high alkalinity of the preparation. It is rather remarkable how tolerant the muscles seem of this drug, as no patient has complained of any pain or stiffness when getting about again, although some of them had had as many as eighteen doses spread over three days in various parts of their vastus externus muscles.

In only two extremely toxic cases was meningococcal serum given intravenously, possibly with benefit. The serum we used (P.D. & Co.), is supposed to be antitoxic as well as bactericidal.

The results of treatment were strikingly beneficial in most cases, as, excluding the mixed pneumococcal case, only five fatalities occurred. Two of these cases were children who were moribund on admission and only survived a few hours. One had been ill for three days and one was a fulminating case which only survived six hours from the observed onset of the symptoms. In this case there was a profuse purpuric rash ; the child was comatose and collapsed when admitted three hours from the onset of symptoms, and made no response to any treatment whatever. It was of interest that this case showed hardly any changes in the cerebro-spinal fluid. There was obviously an overwhelming meningococcal septicæmia.

Another fatal case was a chronic case which had been overlooked, probably of six weeks duration, in a young child with symptoms of hydrocephalus. In only two fatal cases could recovery have been expected. One patient was an infant of eighteen months, who was admitted on the third day, did not respond in the least to treatment, and died three days after admission ; the other was a youth of 19, admitted on the sixth day when signs of hydrocephalus were present, and he died within forty-eight hours.

In cases of severe vomiting death from collapse due to de-hydration is a serious danger. This is best overcome with sub-cutaneous injections of 5 per cent. glucose solution in half-normal saline, in amounts from half to one pint, followed up with rectal saline.

Unlike one's experience in the 1915 epidemic, relapses were conspicuous by their complete absence. One case convalescent from this disease was admitted from another Institution with a diagnosis of diptheria. This case proved to be one of agranulocytic angina, and an examination of this patient's blood showed an almost complete absence of neutrophile leucocytes. The case terminated fatally forth-eight hours after admission. On inquiry it was found that the man, aged 28, had been treated for fourteen days with large doses of sulphopyridine.

Lumbar puncture was only done for diagnostic purposes, as we did not find it of any value in treatment in these cases. Our experience this winter suggests the vital importance of the early and intensive treatment with sulphapyridine if the presence of meningococcal meningitis is suspected. The improvement under the drug is almost incredible to one who has had experience of our former ineffective treatment. Improvement was evident within twenty-four hours of starting the treatment—the intense headache was usually gone within forty-eight hours, and in the comatose cases the restoration of consciousness occurred within the same period. In all the recovered cases the patient was over the disease, free of headache, and perfectly comfortable by the end of four days.

Our mortality rate in this series is 12 per cent. at the moment, compared with 47 per cent. in the last epidemic. The painful chronic cases we used to see, which sometimes dragged on for some weeks or even months, were completely absent in this series.

The examination of the cerebro-spinal fluid was done by the staff of the Preventive Medicine Department of the University of Bristol, but typing of the organisms has not been carried out.

